# Magnetic Phase Transition in Ion-Irradiated Ultrathin CoN Films via Magneto-Optic Faraday Effect

**DOI:** 10.3390/ma6115247

**Published:** 2013-11-15

**Authors:** Chiung-Wu Su, Yen-Chu Chang, Sheng-Chi Chang

**Affiliations:** Department of Electrophysics, National Chiayi University, 300 Syuefu Rd., Chiayi 60004, Taiwan; E-Mails: y.c.chang0620@gmail.com (Y.-C.C.); cloudmiss54@gmail.com (S.-C.C.)

**Keywords:** magneto-optic Faraday effects, Auger electron spectroscopy, cobalt nitrides, magnetic anisotropy, zinc oxide, magnetic phase transformation

## Abstract

The magnetic properties of 1 nm thick in-plane anisotropic Co ultrathin film on ZnO(0001) were investigated through successive 500 eV nitrogen-ion sputtering. Magneto-optical Faraday effects were used to observe the evolution of the ion-irradiated sample in longitudinal and perpendicular magnetic fields. The ferromagnetic phase of the initial in-plane anisotropic fcc β-Co phase transformation to β-Co(N) phase was terminated at paramagnetic CoN*_x_* phase. In-plane anisotropy with weak out-of-plane anisotropy of the Co/ZnO sample was initially observed in the as-grown condition. In the sputtering process, the N^+^ ions induced simultaneous sputtering and doping. An abrupt spin reorientation behavior from in-plane to out-of-plane was found under prolonged sputtering condition. The existence of perpendicular anisotropy measured from the out-of-plane Faraday effect may be attributed to the co-existence of residual β-Co and Co_4_N exchange bonding force by the gradual depletion of Co-N thickness.

## 1. Introduction

Developing renewable, clean energy is an important concern in the 21st century. Hydrogen power generation should be based on some key catalysts, such as ammonia (NH_3_), that provide a reaction route for splitting N and H. Cobalt-based nitrides, such as Co*_x_*N and Co*_x_*Mo*_y_*N, are popularly used as industrial catalysts in ammonia (NH_3_) decompositions [1,2,3]. These nitride materials exhibit excellent chemical and physical properties, including hardness and high melting point. The exploration of catalyst structures and physical properties is advantageous to understand better catalytic processes and advanced applications.

This study is based on the recent studies on CoN*_x_* film composition through different plasma energies of nitrogen [4]. In a fixed plasma energy, we found a distinct composition of saturated CoN*_x_* after ion implantation. The implantation process continues until the composition ratio of Co to N becomes saturated. Then, the depletion of the CoN*_x_* thickness begins with constant ratio until the end of sputtering. CoN*_x_* is also a magnetic material. Therefore, the evolution of magnetic property in the post-nitriding process should be examined. The magneto-optical Faraday effect (MOFE) measurement is used to observe the whole evolution of the magnetic phase transition. The evaluated sample is 1 nm thick Co film deposited on a Zn(0001) crystal. Strong in-plane and weak out-of-plane anisotropies are dominated in the as-grown Co/Zn(0001). The surface anisotropy energy may be retained after prolonged sputtering.

The first-order magnetic phase transformation can be expressed as a function of temperature [5]. Magnetic ordering is always dominant in critical structures in which the magnetic phase of a magnetic system is transformed. Equilibrium and metastable states for magnetic phase affect media performance, such as reversibility and noise control. Thus, structurally induced magnetic properties are attracting attention in advanced spintronics systems, for example, metallic films on metal or semiconductor surfaces [6,7]. Multidiscipline science communities investigate thin nitride films because of their wide potential applications in the fields of ultrahigh-density magnetic recording, catalysis, or even in refractory materials and biosensors [8,9,10,11,12]. FeN has many phases, including the known Fe_16_N_2_ single-crystal phase, Fe_4_N, Fe_3_N, and Fe_2_N [13,14,15,16,17]. The fundamental magnetic property of CoN has been less well explored than that of FeN. Similar to FeN, CoN contains rich chemical phases, including β-Co(N), fcc-Co_4_N, hcp-Co_3_N, orthorhombic-Co_2_N, zinc blende-type, and NaCl-type CoN [18]. The ferromagnetic phases of Co_4_N, the Co_3_N, Co_2_N, and CoN have been characterized as paramagnetic. However, the formation of certain chemical phases, e.g., Co_4_N, is still not clearly elucidated [10,19]. Wang *et al.* [18] recently studied the structural and magnetic properties of DC-sputtered Co-N thin films. Structural phases, for example, β-Co(N), Co_4_N, and Co_3_N, are generated by controlling the sputtering power. Different surface morphologies are observed under scanning electron microscope, and electron diffraction patterns are examined to determine their relation with magnetic properties. However, using the sputtering method to grow ultrathin Co-N films is challenging, especially fabrication at high sputtering powers (~10 W to 200 W). In this study, we attempted to use an ultra-low sputtering power (~0.011 W) by focusing an ion beam with a fluence of 1.3 × 10^14^ ions/cm^2^ s to post-irradiate grown Co/ZnO(0001) film. This paper is focused on the observation of magnetic properties during nitridation.

From our observations, the β-Co(N) phase corresponds to the initial stage of sputtering. Metastable phases with few fractions in N may be retained in the initial implantation. During irradiation, an increase in N fraction resulted in the direct transformation from ferromagnetic Co-rich phase to paramagnetic N-rich phase. After prolonged sputtering, the paramagnetic phase was estimated to be CoN_2.3_, which was ultimately examined by Auger electron spectroscopy (AES). The observation of the nitriding process in CoN*_x_* is conducive to evaluate the unexplored magnetic properties. In the whole process, an evolution of magnetic phase transformation from ferromagnetic to paramagnetic that is directly related to N composition was observed.

## 2. Principle and Experiment

All magneto-optical measurements and processing have been in-situ performed in an ultrahigh vacuum environment with a base pressure of 2 × 10^−^^10^ Torr at room temperature because of the material scale. During Co film deposition, the vacuum was maintained at ~1 × 10^−9^ Torr. The growing rate of the Co film was ~0.5 nm/h. The high transmittance of the Co/ZnO sample in visible spectrum made the magneto-optical Faraday effect suitable for evaluating the magnetic properties during ion irradiation. A complete hysteresis loop can be measured through min polarization rotation, which depends on the increase of the magnetic field. The Faraday effect is a magneto-optical phenomenon similar to Kerr effect, which is widely applied for investigating magnetic thin film system. Generally, Faraday effects can be found in all dielectric materials. However, in the present film/substrate system, the real optical intensity in transmission mode included both signals from the film and substrate. Thus, the principle of magnetic property in experimental determination can be extended according to a similar setup of surface magneto-optical Kerr effect technique in vacuum [20,21]. All contributions in this Faraday magneto-optic effect should be considered in the following merged form:

(1) $${\theta_{F}}^{Total}={\theta_{F}}^{Sub}+{\theta_{F}}^{Film}=V_{F}HL+\rho_{F}\frac{M}{M_{S}}L=\frac{\delta }{4}\cdot \frac{I_{T}-I_{0}}{I_{0}}$$

where the total Faraday effect should include two terms, namely, the dielectric substrate and the magnetic film. Substrate effect from a paramagnetic or a diamagnetic material is dominated by a Verdet constant *V*_F_ (in rad [T m]^−1^) [21]. The coefficient ρ_F_ is the rotatory power or specific Faraday rotation from the magnetic films, and δ is the deviation angle from *s*-polarization, at which the optical intensity in analyzer reaches a minimum value. *L* is the length of the path where light and magnetic field interact. The film terms in Equation (1) are specifically adapted to ferromagnetic materials, which contribute to rectangular hysteresis behavior. The rotation of polarization θ_F_ is totally obtained through one optical detector with crossed polarizers. The total magneto-optical intensity *I*_T_ involves two independent quantities, namely, the substrate optical intensity *I*^Sub^ controlled by the field and film optical intensity *I*^Film^ controlled by the magnetization as shown in the fourth term of Equation (1). Therefore, the magnetization (M) of a magnetic film is only proportional to its contribution in optical difference *I*^Film^ – *I*_0_. Thus, reversed behavior caused by positive and negative rotation in polarization from film or substrate may exist. The coercive force (*H*_C_) is not directly involved in the equation because it should be separated as an independent factor, such as chemical or magnetic structure. A substrate Faraday effect observed in the out-of-plane configuration cannot be omitted. In this study, a paramagnetic-like behavior was observed in all PMOFE data.

**Figure 1:**
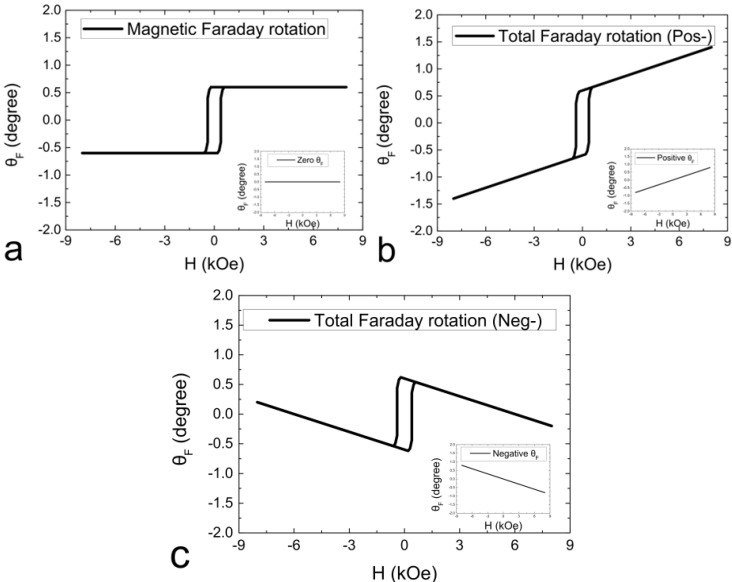
Simulated hysteresis loops of magneto-optic Faraday effects considering the magnetic film signals and different signals from the substrate: (**a**) zero; (**b**) positive; and (**c**) negative rotations.

Figure 1 exhibits the simulation of conditions indicated as zero, positive (a positive background slope in the inlet of Figure 1b), and negative (a negative background slope in the inlet of Figure 1c) Faraday effects predicted from the substrate and a film. The corresponding positive or negative Faraday effect (rotation) is not treated as paramagnetic or diamagnetic phase because of the optically birefringence phenomenon in ZnO(0001). In the magneto-optic measurement, the positive rotation on the deviation angle δ indicated in Equation (1) contributes to the spin-up (↑) state in a positive field. In our case, the δ value was deviated from the *s*-polarization analyzer in a clockwise rotation from the observer in a positive magnetic field.

**Figure 2:**
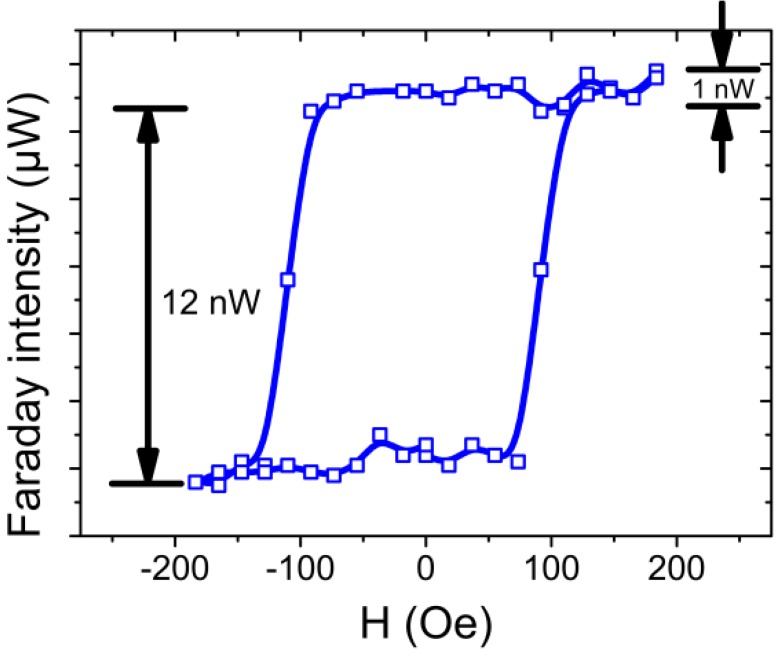
Sensitivity test of the longitudinal magneto-optic Faraday effect measurement for 1.2 nm Co/ZnO(0001). The saturation Faraday rotation θ_F_ converted from the intensity difference of *s*-polarization was approximately 0.074°. The maximum field applied in this loop was set to 190 Oe.

To verify this assumption, we further observed the effect on the undetermined magnetic properties in the longitudinal and perpendicular fields. The field direction in the ultrahigh vacuum chamber was defined elsewhere [22,23]. Figure 2 demonstrates the detection limit of our optical devices. The minimum fluctuation in optical intensity is 1 nW. According to the intensity difference from spin-up to spin-down, the hysteresis example converted to saturation Faraday rotation is approximately 0.074° (~6 nW). The sweeping rate of the magnetic field was ~17 Oe/s, and the maximum field was set at ~500 Oe. The ions have been generated from an ion gun (source model: ISE 10; Omicron NanoTechnology Co., Ltd., Taipei, Taiwan). The ion energy was fixed at 500 eV because the condition of low-energy sputtering led to relatively large N fraction in Co film in previous studies [4]. The initial magneto-optic properties of 1.0 nm Co/ZnO(0001) exhibited strong in-plane anisotropy. The hysteresis loop of ion-irradiated sample was monitored to observe the evolution of magnetic phase after each sputtering by N^+^.

## 3. Results

**Figure 3:**
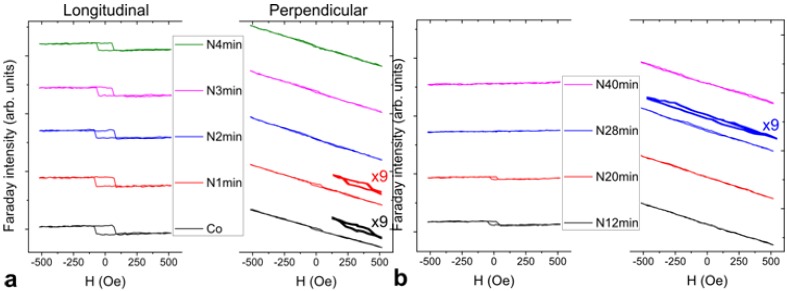
Evolution of post-nitriding hysteresis loops for 1.0 nm Co/ZnO(0001) with sputtering time. Longitudinal (in-plane, L) and perpendicular (out-of-plane, P) field MOFE measurements were divided into (**a**) initial short sputter time interval (1 min); and (**b**) long sputter time interval (8 min to 12 min).

**Figure 4:**
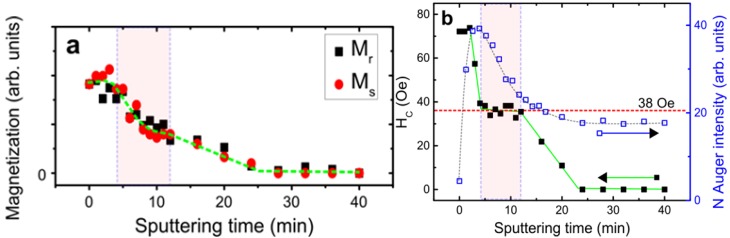
Fundamental magnetic parameters, saturation magnetization, and remanence (in arbitrary units), coercive force (in Oe), and N *KLL* peak height intensity (in arbitrary units) as functions of sputtering time. The shadow region is marked corresponding to the CoN*_x_* compound phase in 500 eV.

Figure 3 shows the evolution of hysteresis loops after each N^+^ sputtering. Longitudinal (L-) and perpendicular (P-) MOFE measurement were studied to observe the variation in magnetic anisotropy. The inverted or mirrored hysteresis loops with respect to the *M*-axis in Figure 3 are due to the reversed field applied to the positive Faraday rotation. Normally, field direction has been determined as counterclockwise Faraday rotation by the right-hand rule. In our case, reversed positive magnetic field corresponds to clockwise Faraday rotation because the initial polarity of electromagnet is reversed. Thus, in the magneto-optic measurement, the spin-down (↓) state in the negative field is inverted at which the analyzer is adjusted clockwise to an angle +δ from minimum polarization intensity. Therefore, the optical intensity caused by Faraday effect decreases with the positive field. From the experimental result, the data were divided into groups with short sputter interval of 1 min in Figure 3a and long sputter interval from 8 min to 12 min in Figure 3b. From the LMOFE results, the film has a strong easy-plane anisotropy close to the plane in the initial 1.0 nm Co/ZnO(0001). The reasonable reduction in coercivity and magnetization values after prolonged sputtering may be misguided to the deposited film purely sputtered from the substrate. In fact, we found that the inference was not true because Co and N still remained on the surface, where the compositions were examined in the Auger spectra determination (Figure 4).

The out-of-plane MOFE signal was also found on the right-hand side of Figure 3. Most signals with a paramagnetic-like behavior come from the substrate. A weak perpendicular anisotropy was found in the initial data for pure Co film (Figure 3a). The hysteresis behavior was clearly observed after 9× zoomed-in amplification. The paramagnetic-like loop is attributed to the total contribution of positive rotation from the substrate and the film in the perpendicular field as mentioned in Figure 1. Instead of the in-plane LMOFE signals, the out-of-plane PMOFE signals disappeared as time passed. Thus, positive surface anisotropy with an out-of-plane component may be dominant in the initial Co/ZnO surface. A decrease in H_C_ in PMOFE was observed after 1 min of sputtering. The increased penetration of N fractions in the Co film gradually altered the original magnetic nature of Co film as shown in the latter LMOFE data. Except for the weakening of magneto-optical signals by sputtering, the loop of LMOFE notably disappeared after 20 min and found an abrupt transition from in-plane to out-of-plane with large H_C_ at ~150 Oe in the reversed loop of PMOFE at the 28th min. The reversed loop was a combination of positive rotation from the substrate and negative rotation from the film as predicted in the third case of Figure 1. In this condition, the thickness has to be greatly reduced by ion sputtering, whereas superparamagnetic phase at the time can be excluded because of the huge H_C_ value. The strong chemical bonding of ultrathin CoN*_x_* in the *c*-axis may be induced during the phase transition [24]. The paramagnetic-like and reversed loops are supposed to be different from chemical bonding, that is, Co-rich to N-rich phase, formed in the post-nitriding process. The reversed behavior of magnetic contribution from the Co-N bonding needs further detailed investigations. However, in this case, excess N fraction simultaneously broke the in-plane and out-of-plane anisotropies of CoN*_x_*, which induced the magnetic phase transformation from ferromagnetic to paramagnetic. The sputtering effect, which dominated a depletion role to the thickness of Co-N, surpassed the implantation effect of N ions into Co after prolonged sputtering. The magnetic domain in this condition may have been lost and could not be detected by MOFEs. Thus, the hysteresis behavior completely disappeared in the 40th min even if the composition of Co and N still existed in further examination by AES.

**Figure 5:**
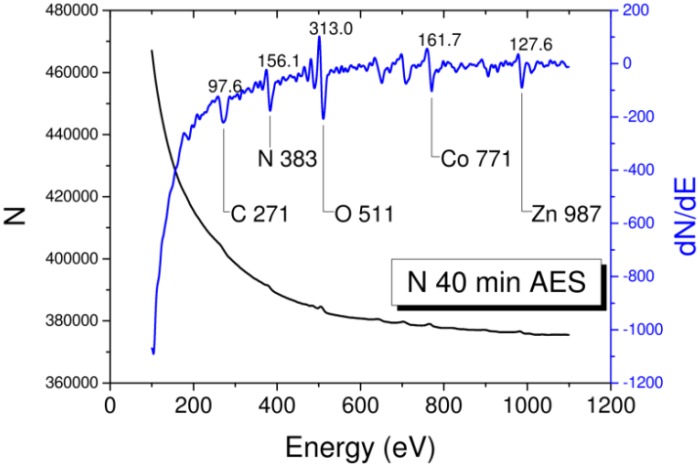
Auger spectrum of the sample after nitriding process was scanned at 40th min. Characteristic Co *LMM*, N *KLL* and substrate Zn *LMM*, O *KLL* with impurity C *KLL* Auger transitions with peak height intensities are all indicated.

Figure 4 demonstrates the saturation magnetization (*M*_s_), remanence (*M*_r_), and *H*_C_ as functions of sputtering time. The sputtering energy at 500 eV refers to the optimum condition by AES investigation [4]. Under optimized condition, the effect of implantation was relatively more evident than that of sputtering compared with other sputtering energies. The nitridation behavior in the variations in magnetic properties (Figure 4a) was consistent with the variation in surface composition (Figure 4b). The initial sputtering of the DC N^+^ plasma before the 4th min corresponds to the absorption of nitrogen on the top of Co film (the increase of N *KLL* Auger peak height intensity). As shown in the marked area in Figure 4b, thickness depletion with an exponential decay in N composition occurred between 4th min and 12th min. The invariant composition ratio CoN*_x_* was retained in this region. The inference is quite reasonable because the coercive force saturates at ~38 Oe (labeled in Figure 4b). A typical sputtering effect of ions on surfaces was discovered between the 12th min and 24th min. Magnetization and H_C_ monotonously decreased with time. The hysteresis loop at the 28th min was reversed with large coercivity in the out-of-plane PMOFE measurement in Figure 3b. Given the data of short time interval in Figure 3a, the film is consistent with the result of β-Co(N) phase with low H_C_ (~34 Oe) [18]. For the long time interval in Figure 3b, the breaking of Co-N bonding led to desorption of N; precipitation of Co may occur. Therefore, the generation of pure β-Co phase accompanied by a paramagnetic CoN*_x_* phase is possible, which is consistent with the inference where the highest sputtering energy is the same as our case at 500 V [18]. The condition implied that ion-induced precipitation or segregation mechanism in prolonged N^+^ irradiation is similar to the SiN:H study [25]. We investigated the coercivity data of *fcc* Co_4_N phase in Ref. [18] at ~100 Oe to 170 Oe in 400 V to 500 V cases, which is close to our value (~150 Oe); weak ferromagnetic phase is measured at the 28th min by PMOFE. The sputtering experiment was finally completed without any magnetic information at the 40th min. No Co or N existed after prolonged sputtering. However, contrary to the original inference, the chemical composition of Co and N was still determined on the surface after the AES examination. No obvious chemical shift for Co *LMM* and N *KLL* Auger peaks was found in Figure 5. Therefore, the possibility of CoN*_x_* compound in this region is very low. Amorphous Co-N or interstitial alloy phase is possible. The thickness at the 40th min was estimated to be 0.46 nm (equivalent to 2.2 monolayer thick of Co and approximately 70% intermixing of interstitial N ions).

## 4. Discussions

Cobalt nitrides have various structural phases. Only β-Co(N) and fcc-Co_4_N belong to the ferromagnetic phase [18]. γ-Co_3_N, δ-Co_2_N, or specifically interstitial compounds, such as Co_3_N_2_ and Co_2_N_3_, have paramagnetic phases [26]. In the ultrathin thickness region, the amorphous structure of CoN*_x_* is paramagnetic because of the spin fluctuation and domain elimination. According to the theoretical study by first-principle calculations, the ultrathin CoN*_x_* film is favored to paramagnetic phase [11]. If the relative fraction of N in CoN*_x_* is too high, the magnetic ordering of CoN*_x_* can be easily transformed to paramagnetic. In this study, we used low sputtering energy (500 eV) to increase the possibility of doping amount and decrease the destruction of sputtering effect to the Co film. Thus, the magnetic phase transition from ferromagnetic to paramagnetic was clearly observed. The phenomenon was first systematically investigated by longitudinal and perpendicular MOFEs. From the previous result of surface composition investigated by AES, three typical evolutions in composition as functions of sputtering time were identified when a distinct thickness of Co ultrathin film (<2 nm) was applied [4]. In the first stage of phase transition, the increase in the N fraction corresponded to the implantation into the Co film. N^+^ absorption was saturated in the second stage of phase transition. A constant composition ratio of Co/N was dependent on the sputtering energy. The ratio of the sample was determined from the initial thickness and sputter energy of the forming CoN*_x_*. The CoN composition in this region could not be altered, whereas the corresponding coercivity value was saturated at ~38 ± 2 Oe. In general, invariant coercivity exists in the same type of magnetic structure. The property of high brittleness of CoN*_x_* is evident in this region (region II in [4]). Therefore, the depletion effect with distinct composition of CoN*_x_* is obvious, which follows an exponential decay behavior on the magnetization in Figure 4. In the third stage of phase transition, the bonding of constant composition of Co and N was broken. The thickness became too thin to maintain the magnetization because of prolonged sputtering. Over 24 min, only few fractions of CoN were embedded on the ZnO surface, and N ions could not be easily desorbed. The N *KLL* Auger signal was ultimately saturated to the end as shown in Figure 4b. Studies on heteroepitaxial growth, such as GaN on sapphire by TEM and on SiC by X-ray rocking curve, have noted that a structural anisotropy is strongly related to stacking faults and defect densities [27,28]. Crystalline with randomly distributed facets induces dominant defect luminescence. The structural-induced crystalline phase of the material may be the key to identifying the detailed magnetic information. However, this short communication focused on the transition of magnetic phases between magnetic properties, and surface compositions seems beyond the scope of this discussion. More experimental investigations on the structural properties and theoretical works are needed despite a big challenge regarding the measurement of ultrathin film system.

## 5. Conclusions

In this study, the magnetic properties of post-nitridation process in Co/ZnO(0001) film were first evaluated in vacuum MOFE technique. The Faraday effect, including substrate signal from ZnO(0001), is clearly observed in the perpendicular magneto-optical Faraday effect. Ferromagnetic to paramagnetic transition was clearly observed in the sputtering process. Weak spin transformation from in-plane to out-of-plane was also observed in the prolonged nitridation. The results indicate that the magnetic phase transformation of CoN*_x_* follows the chemical composition, which is consistent with the variation in the AES results. The ferromagnetic phase is dominant in the initial implantation and further compound formations. The paramagnetic phase exists under nitrogen-rich condition. Paramagnetic CoN*_x_* phase can be easily fabricated using low-energy sputtering.
